# Basic and clinical immunology – 3031. The correlation between serum protemics patterns of sTRAIL and CXCL8 with FDG-PET/CT findings in bevacizumab treated colon cancers

**DOI:** 10.1186/1939-4551-6-S1-P206

**Published:** 2013-04-23

**Authors:** Arzu Didem Yalcin, Atil Bisgin, Aysegul Kargi, Burhan Savas, Metin Erkilic, Antonia Dimitrakopoulou-Strauss, Ludwig Strauss

**Affiliations:** 1 Internal Medicine, Allergy and Immunology, Education and Research Hospital, Turkey; 2 Cancer institue., Sweden; 3 Department of Internal Medicine, Antalya Education and Research Hospital, Antalya, Turkey; 4 Department of Internal Medicine, Oncology Unit, Akdeniz University Hospital, Antalya, Turkey; 5 Department of Nuclear Medicine, Akdeniz University Hospital, Turkey; 6 Clinical Cooperation Unit Nuclear Medicine, German Cancer Research Center, Im Neuenheimer Feld 280, 69120, Heidelberg, Germany

## Background

Bevacizumab is a humanized monoclonal antibody developed against vascular endothelial growth factor (VEGF) for the treatment of metastatic colorectal cancer (MCRC). The changes and correlations of sTRAIL and CXCL8 prior to treatment and three months following therapy as well as the corresponding Positron emission tomography (18FDG-PET/CT) results were evaluated.

## Methods

The measurements were taken before and after treatment for comparison purposes. The study population comprised 17/29 patients with MCRC, undergoing PET/CT scanning prior to treatment. We were able to perform a follow up PET/CT examination three months after onset of therapy in 15/17 patients.Patients were instructed to fast for at least 6h before an injection of 18F-FDG(5 MBq/kg)(GeminiPET/CTsystem). Images were reconstructed using the maximum-likelihood 3-dimensional algorithm according to standard clinical protocol: 2 iterations,relaxation parameter of 0.05,5-mm,3-dimensional gaussian postfiltering, a4·4·4mm-voxel grid sampling, and attenuation correction based on a low-dose CT scan.All images were visually interpreted by consensus between two experienced nuclear physicians and standardized uptake values(SUV_max_) were calculated from the image data.

## Results

There were significant changes prior to treatment and three months later for sTRAIL(p=0.0080) and CXCL8(p=0.0001). Generally, sTRAIL values were increasing during therapy, while a decrease was observed for CXCL8. Correlation analysis was applied to the data and revealed significant correlations for the SUV_max_ in the primary tumor prior to treatment and CXCL8 prior to therapy (p=0.0303). Furthermore, significant correlations were observed for the SUV_max_ and sTRAIL(p=0.0237) as well as CXCL8(p=0.0002) three months after treatment initiation. CXCL8 prior to treatment was also correlated with the SUV three months after onset of treatment(p=0.0072). A significant correlation was noted for one combination of two variables, the SUV_max_ in the metastases and CXCL8 prior to treatment (p=0.0175). These results are supported when we group the SUV_max_ in the metastases following treatment into two groups with SUV_max_<5 and SUV_max_>5. There is a significant difference for both groups regarding overall survival, with a lower survival associated with SUV_max_s exceeding.

## Conclusions

This study provides evidence that proteomics patterns of sTRAIL and CXCL8 predict tumor response und survival in MCRC patients treated with bevacizumab and within a high concordance of 18-FDG-PET/CT findings. The high correlation of CXCL8 and FDG uptake in metastases prior to treatment with survival direct to a promising approach to individualize treatment of patients.

